# Does Brand Attitude Complement Influencer Credibility in Shaping Purchase Intention of Indian GenZ Consumers?

**DOI:** 10.12688/f1000research.157553.1

**Published:** 2024-11-08

**Authors:** Pranav Vilas Chavare, Smitha Nayak, Ramona Birau, Varalakshmi Alapati

**Affiliations:** 1 Department of Computer and Communication Engineering, Manipal Academy of Higher Education, Manipal Institute of Technology, Manipal Karnataka, 576104, India; 2 Department of Humanities and Management, Manipal Academy of Higher Education, Manipal Institute of Technology, Karnataka, 576104, India; 3 Faculty of Economic Science, U, University Constantin Brancusi of Tg-Jiu, Romania, Romania; 4 Department of Commerce, Manipal Academy of Higher Education, Manipal, Karnataka, 576104, India

**Keywords:** influencer credibility, purchase intention, attitude towards brand, India

## Abstract

**Introduction:**

The social media landscape has undergone radical changes and has revolutionized consumer perspectives, purchasing habits, and behaviors. Amidst this emerging trend is the rise of influencer marketing and its impact on the purchase intentions of followers. The objective is to explore the characteristics of influencers that contribute to their credibility. This research aims to explore the role of consumers’ attitude toward brands on their intention to adopt brands endorsed by influencers.

**Methods:**

This cross-sectional research was undertaken among GenZ in the urban landscape of India. Data collected was analyzed using SmartPLS4 software.

**Findings:**

Trust, expertise, and similarity were the significant antecedents of the formation of influencer credibility. Attractiveness did not have a significant influence on influencer credibility. A complementary partial mediation of Attitude towards a brand is observed in the association between influencer credibility and the purchase intention of followers. Attitude towards the video also had a significant positive influence on purchase intention.

**Conclusion:**

The study found that Gen Z places little importance on an influencer’s attractiveness, as it has no significant impact on credibility. However, attitude toward the brand strongly influenced purchase intention and partially mediated the relationship between influencer credibility and purchase intention.

## 1. Introduction

The social media landscape has undergone radical changes over the past few years. It has become an important part of people’s everyday routines and significantly influences cultural aspects of society. It has revolutionized consumer perspectives, purchasing habits, and behaviors ( Y. Dwivedi, 2021). In today’s technology-driven world, social networking platforms have become an avenue where brands can extend their marketing campaigns to a wider range of consumers. Social media has transformed the way businesses connect with their audience, and India is no exception to this digital revolution Social media platforms such as Facebook, YouTube, Instagram, and Twitter have become the grounds for brands to engage with customers, build relationships, and drive sales. India has embraced the Internet, and its digital population has grown rapidly over the past decade, reaching 600 million active Internet users ( www.statista.com/, n.d.). Access to social media has also enhanced the time spent by users on social media daily. A report published by The Indian Institute of Management Ahmedabad on *“New Age Digital Media Consumption: A survey of Social Media, OTT Content Online Gaming”* reported that Indians, on average, spent 194 minutes daily on social media platforms ( The Esya Centre and IIM Ahmedabad, 2023) and most of them were in the age group of 18 to 34 years of age ( GWI, 2023). This forms a strong foothold for brands to emphasize digital marketing strategies to this specific age group. Amidst this emerging trend is the growth of a concept called marketing influencers. Marketing Influencers are defined as “people who have the power to affect the purchase decisions of others due to their real or perceived authority, knowledge, position, or relationship” ( Mavrck, 2014). A further classification of influencers based on the number of followers they have helps different businesses choose which influencers to partner with. While macro-influencers can claim millions of followers, micro-influencers usually have less than 100,000. Micro-influencers are everyday people who post meaningful content with a significant impact on social media sites such as Facebook, Instagram, and YouTube. Social media organizations and celebrities are examples of macro-influencers. In the present digital landscape, consumers seek opinions from their peers (other consumers) and marketing influencers to make informed purchase decisions. Thus, influencers play a major role in shaping consumer purchase decisions and brand choices ( Anjali Chopra and Jaju, 2020).

**Figure 1.**
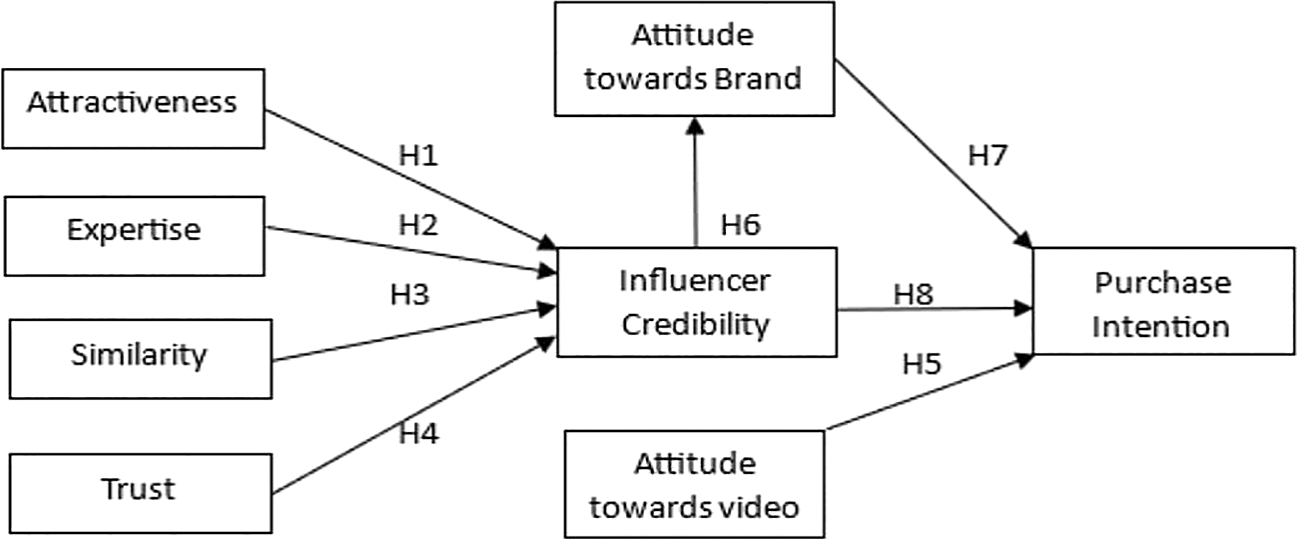
Proposed conceptual framework. Source: Author’s own.

Influencer marketing has been used more recently, but there hasn’t been a single scholarly definition of it—especially when it comes to India ( Johansen and Guldvik, n.d.). Based on the number of followers they have, around 4 million influencers in India are divided into four categories: elite, mega, macro, and micro as of June 2023 ( Katya Naydu, 2023). About 118 million Indians have purchased goods or services that influencers have promoted, according to Roshan Behera (2023). The authors decided to look into the influencer marketing scene in India, specifically from the perspective of Generation Z (those born between 1997 and 2012), given the growing importance of influencers in the country and the paucity of scholarly research on the subject. Further, on social media, Gen Z users are more likely to be drawn to content about fashion, travel, and movies. According to a demographic comparison, millennials follow 52% of Instagram influencers, whereas Gen Z members follow about 97.9% of them ( BrandWagon Online, 2023). This highlights the importance of influencer marketing, especially when aimed at the Gen Z demographic in India. Although the influencers themselves have a charm factor, an important question is what makes their content truly appealing. The answer to this question is perhaps the relationship between the influencer’s credibility, the audience’s views towards the brand, and their attitude toward the content. This study aims to understand the connection between these factors and how the audiences’ attitudes mediate the influencers’ credibility and purchase intention. The objective is to analyze the influencer marketing sector in India and find insights into the characteristics of influencers that contribute to their credibility. Furthermore, study the mediating effect of the audiences’ attitude toward the brand on the relationship between influencer credibility and purchase intention. The proposed conceptual framework is presented in Figure 1.

The subsequent sections of the manuscript are structured as follows: the hypothesis development section presents literature related to the context of the present research endeavor and aids in conceptualizing the proposed conceptual model. The methodology section highlights the study setting, sampling procedure, and the research instrument development process. The results section presents the data analysis, followed by the discussion section and theoretical and managerial implications. The last section of the manuscript presents directions for future research and the limitations of the study.

## 2. Literature review & hypotheses development

### 2.1 Attractiveness

Research reveals a positive link between the perceived attractiveness of a social media influencer and consumers’ likelihood to purchase the advertised cosmetic product. This attractiveness-purchase intention connection likely stems from several factors: grabbing attention, triggering positive emotions, creating social influence, and boosting perceived trust in the influencer’s opinion. However, the strength of this link can vary. Younger consumers or those who highly value physical appearance might be more susceptible to attractiveness pull. Hence it would be worth exploring this association between the attractiveness of the influencer and

H1: Attractiveness of the social media influencers has a significant positive impact on the credibility of the influencer.

### 2.2 Expertise

The capacity of a communicator to make accurate representations in a certain field of knowledge is referred to as expertise ( Anthony R. Pratkanis, 2011). It includes knowledge, understanding, and experience gained from ongoing work in the same profession. A communicator must have in-depth knowledge, applicable abilities, or a respectable title that establishes credibility in their chosen field to be acknowledged as an expert ( Teresa Borges-Tiago et al., 2023). Consumers are more likely to buy a product endorsed by an influencer they perceive as knowledgeable and experienced, suggesting expertise builds trust and guides informed buying decisions. This knowledge-driven trust stems from several factors: reduced purchase risk due to the influencer’s proven experience, empowered decision-making through clear product explanations, and a boost in overall credibility based on their expertise ( Ashraf et al., 2023; Masuda et al., 2022). However, the strength of this connection can vary depending on factors like product type (expertise’s impact is stronger for complex products), target audience (consumers valuing knowledge are more influenced), and influencer niche (expertise relevant to the product matters most) ( Saima & Khan, 2020). So, expertise strengthens influencer credibility thereby converting likings into purchases. Understanding these dynamics among the GenZ followers would be worth exploring as GenZ is believed to be significantly influenced by peer reviews ( Costa E Silva et al., 2017).

H2: The expertise of the Influencer has a significant impact on the Credibility of the Influencer.

### 2.3 Similarity

Similarity implies the extent to which the audience resembles the communicator ( McCracken, 1989). A study was undertaken by Naderer et al. (2021) Documented that similarity with the influencer had a direct positive effect on the purchase intention of the promoted brand. This means that when the influencer showed a brand and a disclosure, it increased the participants’ intention to buy the product compared to not seeing any brand presentation. Furthermore, the researchers found that the trustworthiness of the influencer played a significant role in mediating the relationship between similarity and purchase intention. This is because brand information from someone similar to you is usually regarded as more genuine and credible than, say, a message from the business itself ( Willemsen et al., 2012).

H3: Similarity of the Influencer with the target audience has a significant positive influence on the credibility of the influencer.

### 2.4 Trustworthiness

Trust represents an individual’s belief or willingness to depend upon, an attitude-related object or phenomenon ( Ki et al., 2023). Trust in the context of influencer marketing can be defined as the influencer’s audience’s belief that the influencer is honest, transparent and genuinely supports the endorsed brand. An influencer can enhance their trust in the audience by being authentic in their content and interactions. In the absence of physical interaction, trust becomes a cornerstone for customers to establish trust in online activities ( Gibreel et al., 2018; Ventre & Kolbe, 2020). The audience is more likely to engage and show an enhanced purchase intention when they trust the parties involved. This is applicable in social media, e-commerce, online reviews, etc. ( Ventre & Kolbe, 2020). Confirmed the positive relationship between trust and purchase intention in the context of online reviews. Overall, trust is an important factor contributing to the success of influencer marketing ( Sokolova & Kefi, 2020).

H4: Trust is a significant positive antecedent of Influencer credibility.

### 2.5 Attitude toward video

Greer (2003) found that people’s opinions about the stories they read are influenced by their perception of the reliability of the material they get online. When it comes to health communication, people’s opinions on online health information are greatly influenced by the websites’ perceived legitimacy ( Wang et al., 2008). In the case of advertising, the literature currently in publication indicates a favorable relationship between customer perceptions of the trustworthiness of endorsers and their endorsements ( Goldsmith et al., 2000). It was established that a favorable correlation between attitudes toward website sponsors and their perceived credibility. According to research by Long and Chiagouris (2006), website users’ opinions of a nonprofit organization are greatly influenced by their perception of the website’s legitimacy. Hence in the digital space, it would be worthwhile to explore if the attitude towards the video of the social media influencer influences the purchase intention of their followers.

H5: Attitude towards the video has a positive influence on purchase intention.

### 2.6 Attitude towards Brand

An attitude-towards-the-ad model was developed by McCracken (1989). These factors influence consumers’ perceptions of advertisements. The empirical study’s conclusions imply that consumers’ perceptions of advertisers and advertising are positively impacted by their perceived believability ( Aaker et al., 2012). Established that admired brands motivate consumers to purchase more from the brand. According to Roberts (2005) Brands gain the respect of consumers by performing well, which eventually leads to the development of a positive reputation. According to a research project by Trivedi & Sama (2020), consumer-favorite brands enjoy favorable relationships with their customers. Therefore, it may be necessary to investigate the extent to which brand attitude influences followers of social media influencers’ purchase intentions directly and through mediation.

H6: Influencer credibility has a positive influence on Attitude toward the brand.

H7: Attitude toward the brand positively influences purchase intention.

H9: Attitude toward brand mediates the association between influencer credibility and purchase intention.

### 2.7 Influencer credibility

Influencers on social media are essential for improving marketing campaigns in the digital era. Choosing influencers wisely about the target market can have a big impact on audience purchase intentions and increase brand recognition. Purchase intention, according to J. P. Trivedi (2018) and Shah and Pillai (2012), arises from consumers’ comprehension of the thinking behind the choices they make regarding brands ( Ki et al., 2023). Claim that it illustrates a scenario in which consumers are likely to purchase a product in a given setting. By using influencers in their marketing campaigns, marketers can positively affect the brand by strengthening the image the influencers have established and enhancing product recognition. Customers are more likely to purchase the promoted products when influencers with a strong connection to the target market are used ( Lim et al., 2017). This finding aligns with a study by Singh and Banerjee (2018). Which emphasizes the ability of influencers to attract clients and stimulate purchase intentions through effective communication with their audience. Hence, this research endeavor intends to explore the effect of influencer credibility on the purchase intention of the followers.

H8: Influencer Credibility has a significant positive influence on the purchase intention of followers.

## 3. Methods

### 3.1 Study design

The quantitative research methodology used in this study is called “cross-sectional research design.” “Post-positivism,” which emphasizes the development of empirical tools to interpret and evaluate human behavior, is the fundamental research philosophy. The goal of the inquiry is to investigate the links that are described in the conceptual framework between independent, dependent, and mediating factors. This marketing study was exempt from submission to the Institutional Ethics Committee of Manipal Academy of Higher Education, by clause 6 of the circular titled “Project Exemption from Submission to IEC,” issued by the Institutional Ethics Committee on 14/01/2021.

### 3.2 Study setting & eligibility criteria

A study undertaken by BrandWagon Online (2023) indicated that the Indian social media landscape, especially on Instagram, is cluttered by the Gen Z segment of the population, and 97.9 percent of them follow influencers. Hence, respondents for this study are restricted to Gen Z of the Indian Population. Additionally, results reported by Statista on “Use of social media platforms in India” read that 59 percent of the rural population in India did not use social media platforms and the same figure stood at 33 percent for the urban population. It is also revealed that 28 percent of the urban regions accessed at least 6 social media applications. Hence, this study is restricted to respondents from the urban regions of India only. The third inclusion criterion to respond to the questionnaire was that they had to follow influencers on social media platforms and have purchased at least one brand in the last 6 months endorsed by the influencer. These three inclusion criteria ensured that the response was obtained from a well-focused segment. A Google form was created to capture data from respondents. The respondents were contacted directly by the researchers, who additionally on the study’s objectives and guaranteed that their information would only be used for academic purposes. As a result, the participants were given access to the Google form, which they were asked to fill out and submit online. Data collection was undertaken from November 2023 to January 2024.

### 3.3 Data measurement

A structured questionnaire was designed to collect data from the respondents. Scales to measure the underlying constructs {Purchase Intention ( Mahrous & Abdelmaaboud, 2017); Expertise ( Spake & Megehee, 2010); Attractiveness ( A. Dwivedi et al., 2015); Similarity ( Casaló et al., 2020); Trust ( Mahrous & Abdelmaaboud, 2017); Credibility ( Xiao et al., 2018); Attitude towards Brand ( Spears & Singh, 2004); Attitude Toward Advertisement ( Voss et al., 2003)} were adapted and measured on 5 5-point Likert scale. The questionnaire consisted of 29 questions including the inclusion criteria & demographic details. A Google form was generated and circulated to capture their response from the identified segment of respondents. It was informed to them that the data collected would be kept confidential and would be utilized only for academic purposes.

### 3.4 Sample size

This study adopted a purposive sampling technique. Data was collected from students of Manipal Academy of Higher Education, and the students are the most representative of the target audience for this research. The sample size was estimated based on the number of indicators in the study by ten ( Sarstedt et al., 2017) ie: 29 *10 = 280. It further rounded off to 300. Finally, data was collected from 300 respondents.

### 3.5 Statistical methods

Microsoft Excel (RRID:SCR_016137 available at https://www.microsoft.com/en-gb/) was used to undertake descriptive analysis and Smart PLS software (SmartPLS, RRID: SCR_022040) was used to analyze the underlying constructs of the study and assess the mediation analysis. The Partial Least Square Structural Equation Modelling technique was used to test the additive and linear models.

### 3.6 Pilot testing

Before the completed questionnaire was sent out to participants to undertake a pilot study, two experienced researchers reviewed it to ensure face validity. The input gathered in this approach helped to improve the instrument’s readability. Further, to ensure the internal consistency of the scales, a pilot study was undertaken with 40sample response data. Cronbach alpha of all constructs of the study was above o.7 (Attitude towards brand = 0.956; Attitude towards video 0.938; Purchase Intention = 0.845; Attractiveness of Influencer = 0.777; Credibility = 0.934; Influencer Experience = 0.882; trust = 0.826) except of Similarity with the Influencer was below the threshold value of 0.7. In social science research, a construct with a Cronbach alpha above 0.6 in a pilot study could be retained as it is expected the increase with an increment in sample size. Hence the research team decided to retain the construct and explore it further during the final data analysis phase.

## 4. Results

To investigate the proposed correlations, we used maximum-likelihood estimation in structural equation modeling (SEM). According to Hair (2009) SEM was selected because of its capacity to evaluate the relationships between latent and observable variables directly.

**Figure 2.**
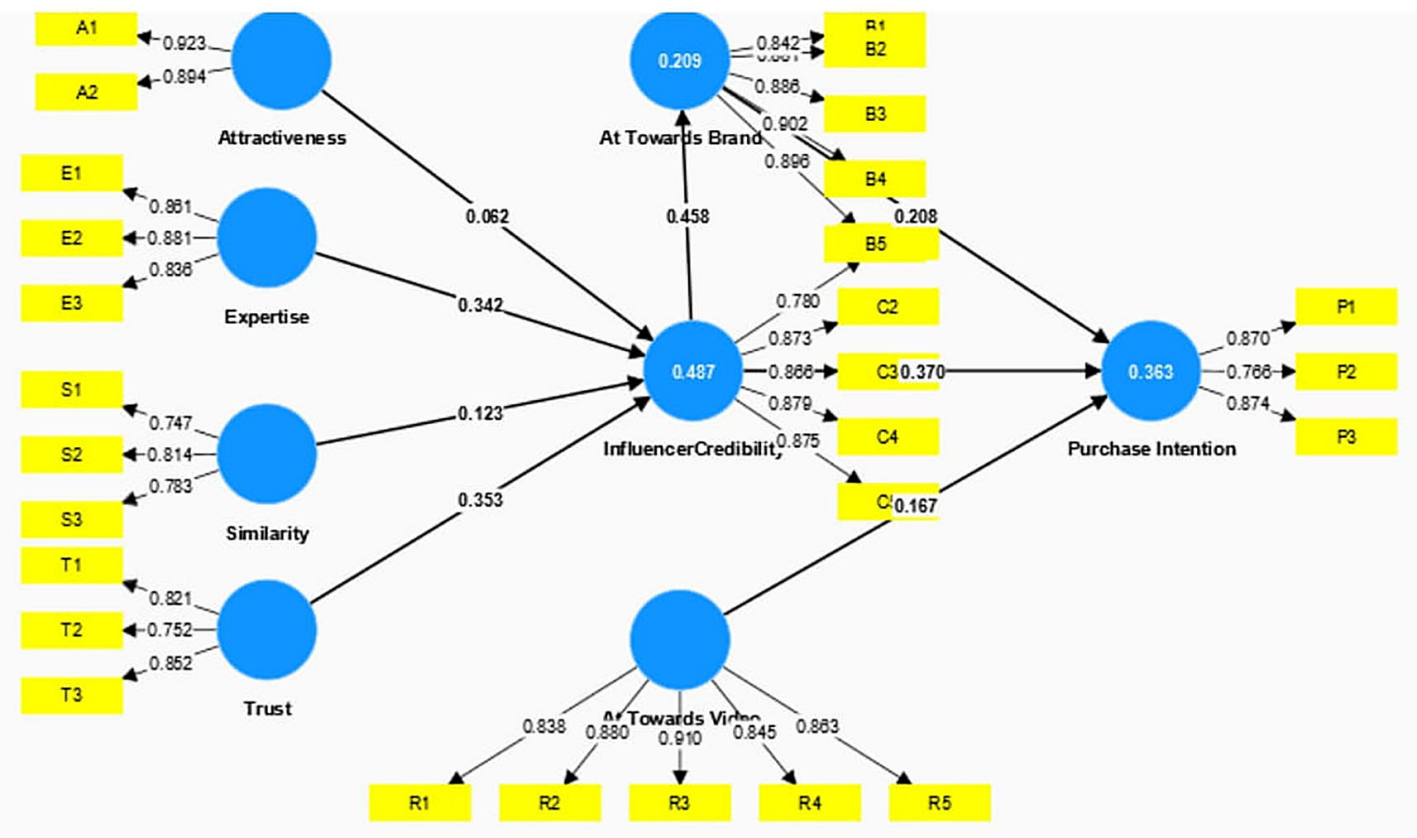
Structural Model.

The construct’s reliability and validity were established by assessing Cronbach’s alpha, rhoa, and composite reliability, as outlined in Table 1. The computed rhoa value surpassing the 0.7 cutoffs indicates ( Hair et al., 2010) robust reliability. Composite reliability is adopted to examine the internal consistency of the constructs and all values are above the threshold limit ( Werts et al., 1974). Cronbach alpha estimates reveal reliability of the all the indicators as all figures were above the threshold value of 0.7 ( Ken Kwong-Kay Wong, 2013). Convergent validity was established using Average Variance Extracted (AVE). All constructs have an AVE above the threshold of 0.5 ( Richard P. Bagozzi and Youjae Yi, 1988). Collinearity among the indicators was examined in the VIF estimates of the constructs. All the VIF values were less than 0.5 ( Sarstedt et al., 2017) no collinearity among the constructs of the study. Similarly, the absence of collinearity among the constructs of the study was established where the VIF values of the inner model of all the exogenous constructs were less than 3.3 ( Joseph F. Hair et al., 2017). This also ensures the absence of common method bias impacting the data ( Kock, 2015). Evaluation of the Structure Model is presented in Figure 2. Indicating Discriminant validity was confirmed using the Fornell and Larcker methodology, as depicted in Table 2.

**Table 1.** Evaluation of structural model.

Construct	Indicators	Outer loadings	AVE	Cronbach Alpha	rho_a	rho_c	Outer weights	VIF
Attitude towards Brand (AB)	AB1	0.842 ***	0.777	0.928	0.930	0.946	0.233 ***	1.746
	AB2	0.881 ***					0.210 ***	1.746
	AB3	0.886 ***					0.215 ***	2.341
	AB4	0.902 ***					0.240 ***	3.180
	AB5	0.896 ***					0.236 ***	3.322
Attractive ness (AT)	AT1	0.923 ***	0.826	0.791	0.803	0.905	0.491 ***	3.300
	AT2	0.894 ***					0.408 ***	3.214
Attitude Towards Video (AV)	AV1	0.838 ***	0.752	0.918	0.934	0.938	0.188 ***	1.902
	AV2	0.880 ***					0.193 ***	2.713
	AV3	0.910 ***					0.281 ***	2.702
	AV4	0.845 ***					0.266 ***	2.846
	AV5	0.863 ***					0.223 ***	2.954
Credibility (C)	C1	0.780 ***	0.732	0.908	0.912	0.932	0.205 ***	1.890
	C2	0.873 ***					0.244 ***	2.111
	C3	0.866 ***					0.231 ***	1.703
	C4	0.879 ***					0.252 ***	1.675
	C5	0.875 ***					0.236 ***	1.568
Expertise €	E1	0.861 ***	0.739	0.823	0.823	0.895	0.397 ***	1.901
	E2	0.881 ***					0.385 ***	2.578
	E3	0.836 ***					0.382 ***	3.181
Purchase Intention (PI)	PI1	0.870 ***	0.703	0.792	0.826	0.876	0.471 ***	3.158
	PI2	0.766 ***					0.295 ***	2.325
	PI3	0.874 ***					0.416 ***	2.875
Similarity (S)	S1	0.747 ***	0.611	0.683	0.687	0.825	0.391 ***	1.343
	S2	0.814 ***					0.413 ***	1.494
	S3	0.783 ***					0.475 ***	1.264
Trust (T)	T1	0.821 ***	0.656	0.743	0.772	0.851	0.433 ***	1.474
	T2	0.752 ***					0.302 ***	1.467
	T3	0.852 ***					0.490 ***	1.488

*** p<0.01.

**Table 2.** Discriminant validity was confirmed using the Fornell and Larcker method.

Constructs	AB	AT	AV	C	E	PI	S	T
**AB**	**0.882**							
**AT**	0.337	**0.909**						
**AV**	0.515	0.396	**0.867**					
**C**	0.458	0.362	0.386	**0.856**				
**E**	0.301	0.288	0.319	0.567	**0.860**			
**PI**	0.464	0.430	0.417	0.530	0.383	**0.838**		
**S**	0.341	0.439	0.401	0.487	0.419	0.536	**0.782**	
**T**	0.512	0.420	0.404	0.598	0.443	0.607	0.551	**0.810**

Subsequently, the structural model was assessed by the bootstrapping technique and the hypothesis was tested. The structural wall was further evaluated with the help of the path coefficients ( Table 3). R ^2^ and Q ^2^ were obtained from the structural model. R ^2^ indicates that a model’s prediction ability should be more than 0.1 ( Falk and Miller, 1992). Trust, Expertise, Attractiveness, and similarity explained 48.7 percent of the variance for Influencer credibility. The credibility of the influencer explained a 20.9 percent variance of Attitude towards Brand and both these constructs explained a 36.6 percent variance of the outcome construct Purchase Intention. Furthermore, Q ^2^, which assesses the model’s predictive usefulness, ought to be greater than 0. The efficiency of the model was confirmed in the current investigation when both R ^2^ and Q ^2^ values were above the designated thresholds, thus the model’s predictive relevance was justified.

**Table 3.** Testing direct relationships.

Hypothesis	Path coefficient	Standard deviation (STDEV)	t value (bootstrap)	P values	F ^2^	BI	Status
H1: At -> C	0.062	0.054	1.135	0.256	0.006	0.043, 0.169	Not supported
H2:E -> C	0.342 ***	0.049	7.007	0.000	0.172	0.245, 0.437	Supported
H3: S -> C	0.123 *	0.059	2.095	0.036	0.018	0.011, 0.239	Supported
H4:T -> C	0.353 ***	0.054	6.549	0.000	0.149	0.245, 0.453	Supported
H5:ATV->PI	0.167 ***	0.060	2.771	0.006	0.031	0.052, 0.286	Supported
H6:C-> ATB	0.458 ***	0.050	9.063	0.000	0.265	0.357, 0.554	Supported
H7:ATB-> PI	0.208 ***	0.056	3.697	0.000	0.045	0.099, 0.320	Supported
H8: C ->PI	0.370 ***	0.060	6.134	0.000	0.164	0.250, 0.484	Supported
R ^2^ AB = 0.209	Q ^2^ AB = 0.208						
R ^2^ C = 0.487	Q ^2^ C = 0.465						
R ^2^ PI = 0.363	Q ^2^ PI = 0.353						

Abbreviation: BI = bias-corrected.

*** p<0.01.

** p<0.05.

* p<0.1.

The results revealed that two exogenous constructs, Expertise (β = 0.342, t = 7.007 p = 0.000) & Trust (β = 0.353, t = 6.549, p = 0.000) had a significant positive impact on the credibility of the influencer. However, Similarity (β = 0.123, t = 2.095, p = 0.036) partially influenced the influencer credibility. Hypothesis 5 (Attitude towards Brand on Purchase Intention) and Credibility of Influencer on Attitude towards Brand were supported revealing two dimensions. Firstly, Attitude towards the Video (β = 0.167, t = 2.771, p = 0.006) has a significant positive association with to Purchase Intention of the followers and secondly, Credibility of the Influencer (β = 0.458, t = 9.063, p = 0.000) had a significant positive influence on the Followers Attitude towards the Brand. Attitude towards Brand (β = 0.208, t = 3.697, p = 0.000) and Credibility of the Influencer (β = 0.370, t = 6.134, p = 0.000) had a significant positive impact on the Purchase intention of the follower. Thus, H7 and H8 were also supported.

### 4.1 Mediating role of attitude towards brand

A mediation analysis was performed to assess the mediating effect of ATB on the linkage between Credibility and PI of the followers. The results ( Table 4) revealed that the total effect of Credibility on PI was significant (β = 0.524, t = 10.305, p = 0.000). With the inclusion of the mediating variable.

**Table 4.** Mediation analysis.

Path	Direct effect, p-value	Total effect, p-value	Specific indirect effect	SD	T Value	p Value	BI (2.5%, 97.5%)	Mediation
H9: Credibility>Attitude towards Brand-> Purchase Intention	0.395, 0.000	0.524, 0.000	0.129, 0.000	0.031	4.201	0.000	0.077, 0.198	Complementary Partial

The Standard Root Mean Square Residual (SRMR), with a suggested threshold of <0.8, was used to assess model fitness. ( Sarstedt et al., 2017). The model’s SRMR rating of 0.055 indicated that it suited the data quite well. Additionally, Q ^2^ was calculated to determine predictive relevance when the endogenous construct was greater than zero.

### 4.2 Importance Performance Matrix Analysis (IPMA)

**Figure 3.**
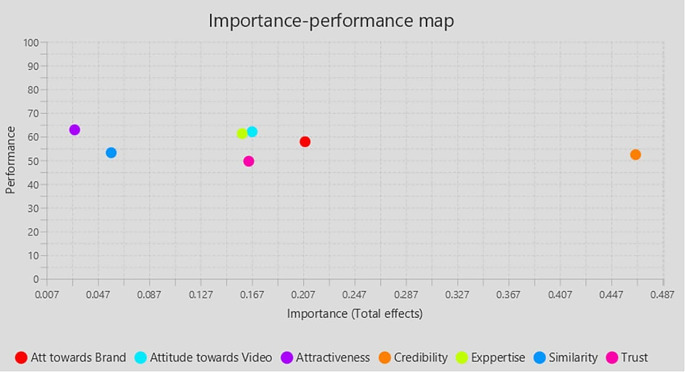
Importance performance matrix.

IPMA’s result is presented as bootstrapping with 5,000 subsamples, demonstrating that all requirements for applying the IPMA approach are met and that some path coefficients are statistically significant. IPMA clarifies the comparative importance and effectiveness of exogenous (Trust, Expertise, Similarity, Attractiveness, Attitude towards Brand, Attitude towards the video, Influencers Credibility) on the endogenous construct (Purchase Intention). Their significance and efficacy are shown by their aggregate impacts and index values. The impact on the endogenous variables is significant. The intrinsic potential of the latent variable scores is demonstrated through effectiveness ( Table 5, Figure 3).

**Table 5.** Important-Performance Matrix Analysis of Behavior Intention.

Latent construct	Importance (Total effect)	Performance (Index value)
Att towards Brand	0.208	57.862
Attractiveness	0.029	62.887
Attitude towards Video	0.167	62.067
Credibility	0.466	52.412
Experience	0.159	61.272
Similarity	0.057	53.226
Trust	0.164	49.631

## 5. Discussion

Influencer marketing has become a potent catalyst propelling brands’ social media investment in the ever-changing world of digital marketing. Influencers provide organizations with a means of accessing pre-existing communities and fostering genuine interactions with their target audiences. Influencers are frequently people with sizable and devoted online followings. Upholding the model proposed by Hovland et al. (1953) on Communication and persuasion, trust in the influencer significantly influences the credibility of the influencer. Similarity with the influencer and Expertise of the influencer is explored to have a significant role in determining the influencer’s Credibility. These findings are in line with the previously published literature on the construct of trust in the marketing influencer ( Johnsson, 2023; Saima & Khan, 2020; Trivedi & Sama, 2020; Patmawati & Miswanto, 2022). Digital media platforms provide internet users with substantial control over the information they share, which fosters a climate even conducive to the spread of misleading information, such as fake news. Internet users now place more emphasis on original information and reliable sources, highlighting the significance of real and honest information sources in the digital sphere.

Further Naderer et al., (2021) documented that similarity with the influencer had a direct positive effect on the purchase intention of the promoted brand. Additionally, a qualitative study undertaken by Johnsson (2023). Among GenZ of Bangladesh, it was documented that most of the followers compared themselves with celebrity influencers and tried to imitate them. This is not justified in our research context as attractiveness did not significantly influence the credibility of the influencer. Another study was undertaken by Trivedi (2018) revealed a higher efficacy of attractiveness the social media influencers. Although the findings contradict the research findings of Johnsson (2023) and Trivedi (2018) revealed, it is noteworthy to observe that these studies are undertaken in different contexts. Trivedi (2018) documented research in the context of fashion influencers. However, it is also documented by Masuda et al. (2022) that the strength of this association can vary depending on the age group of the followers and the product category under consideration. Younger consumers or those who highly value physical appearance might be more susceptible to attractiveness pull. Similarly, luxury or aspirational products can have a stronger effect.

Attitude towards the Video content is documented to have a significant direct positive influence on purchase intention by the followers, leading to acceptance of H5. This finding is also affirmed by Trivedi (2018) that attitude towards the brand endorsed plays a significant role in determining the consumers’ purchase intention. This finding was significantly endorsed in the fashion marketing landscape. This strongly affirms the Theory of Reasoned Action ( Icek Ajzen, 1980).

Attitude towards the brand significantly influenced Purchase Intention and it partially mediated the association between influencer credibility and purchase intention leading to acceptance of H7 and H9. The result is consistent with previous research, supporting the important role that attitudes are shaped by perceived information credibility, as has been shown in studies by Masuda et al. (2022); Naderer et al., (2021) and Wang et al. (2008). This finding also raises questions about the possible value of using YouTube videos’ perceived legitimacy to forecast viewers’ purchase intentions for the products they showcase. The substantial correlation between attitudes and behavioral intentions, a link that Ajzen and Fishbein (1977). Thoroughly examined gives rise to this assumption.

## 6. Theoretical and managerial implications

This study is meaningful in its input to the theoretical understanding of using the Theory of Reasoned Action and the Tri Component Attitude Model in explaining the impact of relevant cues on influencer credibility and purchase intention. This research attempts to deepen knowledge of how GenZ influencer marketing in the context of the Indian market. This study closes a gap in the literature by examining the influence of social media influencers on purchase intentions in India, a topic that has not been well studied. By providing a thorough framework that clarifies the role of influencer qualities in influencing customer purchase intentions through the mediation of credibility, it advances our understanding of the subject. The importance of antecedents of the credibility of the influencer, namely expertise, trust, and similarity, has emerged in this research endeavor. The impact of influencer credibility on purchase intention is well documented and resonates outcomes of previous researchers. This study establishes the significance of the truthfulness and resemblance of the influencer to be effective with his or her followers. It is observed that the target segment (GenZ) gives less importance to the Attractiveness of the influencer and it was insignificant in influencing the credibility. In the backdrop of the Tri Component Attitude Model ( Rosenberg et al., 1960), this finding emphasizes the cognitive component of attitude formation. The Tripartite model, also recognized as the three components of attitude, encompasses affective, behavioral, and cognitive elements. Collectively, these elements create attitudes, with one element usually predominating over the others at any one time. The findings revealed that Attitude towards the brand has a significant positive influence on Purchase intention. The IPMA results also indicate that Attitude towards the brand is the second most important factor determining the purchase intention of followers. Similar previous studies ( J. P. Trivedi, 2018; J. Trivedi & Sama, 2020) have also highlighted the importance of attitude towards the brand in followers’ purchase intention. This strengthens our understanding of the need to build brands and consider influencers as mere enablers of decision-making. This is further validated by the mediation analysis performed in this study. A partial mediation is proved ( Table 4) which indicates that both the antecedents (attitude towards brand and influencer Credibility) have an important role to play in shaping followers’ purchase intention. The significant and positive impact of trustworthiness, similarity, and expertise on influencer credibility should inform advertisers or marketers to use trustworthiness as a criterion to evaluate the power of a social media influencer as a potential advertising partner. This project is also among one of the few studies to analyze the follower’s attitude towards video content on purchase intention. It indicates marketers to take special cognizance of this fact and encourages them to create defined standard operating procedures to be necessarily followed by the influencers. If social media influencer intends to continue their collaborations with advertisers, or wish to expand their followers they must connect with the followers.

## 7. Future directions and limitations

There are certain drawbacks to this study. It doesn’t distinguish between different kinds of videos or brands/products; instead, it concentrates on social media influencers and brands/products in a general way. Subsequent investigations ought to improve the accuracy of influencer marketing theories by examining particular video categories (e.g., office supplies, alcoholic beverages, gaming videos), alternative content formats (e.g., blog posts, audio podcasts, Instagram/Facebook photos), or product genres (e.g., product reviews, how-to tutorials, gaming videos). Furthermore, examining particular groups of micro or macro influencers may reveal differences in the ways that trustworthiness is influenced. Additionally, this study uses a single survey instrument and depends on a single source of consumers, which could introduce a single-source bias. To get over this restriction, future research should think about selecting participants from a variety of sources and using a range of techniques to look more deeply into the dynamics of social media influencer marketing and how consumers perceive the reliability of information.

### Ethical approval and consent

This marketing study was exempt from submission to the Institutional Ethics Committee of Manipal Academy of Higher Education, by clause 6 of the circular titled “Project Exemption from Submission to IEC,” issued by the Institutional Ethics Committee on 14/01/2021.

### Consent statement

No minors were included in the study. It clearly states that while collecting data, respondents were briefed, and then handed over the Google form. The first section of the Google form briefed the respondent again and only if they consent, they clicked the link and presented their consent.

*Consent was taken Verbally and while filling questionnaire the following text was displayed first:*

*Dear Respondent, Marketing Influencers have become a part of our digital ecosystem.*

*In light of this pervasive influencer content that you actively follow, please address the following questions, reflecting on your exposure to these influencers and their messages. Your responses will be kept confidential. If you consent to provide data on your experience with marketing influencer content, click on the link below to proceed. Thank You"*

*The respondent has to consent before clicking on the questionnaire link. This is now has to be how consent was captured from each respondent.*

## Author contributions

• Study conception and design: Prof Smitha Nayak, Pranav Chavare, Dr Ramon Birau
• Data collection: Prof Smitha Nayak, Pranav Chavare,
• Analysis and interpretation of results: Prof Smitha Nayak, Pranav Chavare, Dr Varalakshmi Alapati
• Manuscript preparation. Prof Smitha Nayak, Pranav Chavare, Dr Ramona Birau, Dr Varalakshmi Alapati

## Data Availability

Figshare: [Influencer Marketing [ https://doi.org/10.6084/m9.figshare.27094330.v1] The project contains the following underlying data:
• Impact of Marketing Influencers Dataset Data are available under the terms of the Creative Commons Attribution 4.0 International license (CC-BY 4.0).
