# Supplementary material for: Does Brand Attitude Complement Influencer Credibility in Shaping Purchase Intention of Indian GenZ Consumers?
Source: F1000Res. 2025 Jan 27;13:1343. Originally published 2024 Nov 8. [Version 3] doi: 10.12688/f1000research.157553.3 (PMC11795352; doi:10.12688/f1000research.157553.3)
Supplement: Peer review history

# Reviewer report: Reviewer response for version 3

**DOI:** 10.5256/f1000research.177412.r367236

**Published:** 2025-02-18

**Authors:** Harjinder Kaur Balbir Singh

**Affiliations:** 1 Asia Pacific University of Technology and Innovation, Kuala Lumpur, Malaysia

1. **Introduction**

- **Strengths:** The introduction provides a comprehensive overview of the topic and sets the stage for the research. It effectively highlights the importance of social media and influencer marketing in India.
- **Areas for Improvement:** The introduction is somewhat lengthy. Consider condensing it to focus on the most critical points. Additionally, the transition between different ideas could be smoother to enhance readability. Suggest adding in the specific industry of the study in the title.

**2.Literature Review and Hypotheses Development**

- **Strengths:** The literature review is thorough and covers a wide range of relevant studies. The hypotheses are clearly stated and logically derived from the literature.
- **Areas for Improvement:** Some sections of the literature review read more like summaries rather than critical evaluations. Aim to provide more critical analysis of the studies reviewed. Additionally, ensure that each hypothesis is explicitly linked to the literature discussed. Better to have latest scholarly journals of 5 years data.

**3. Methodology**

**Study Design:**

- **Strengths:** The cross-sectional research design is appropriate for the study. The explanation of the research philosophy is clear.
- **Areas for Improvement:** The explanation of the research philosophy could be more concise. Some sections are overly detailed and could be streamlined.

**Study Setting and Eligibility Criteria:**

- **Strengths:** The criteria for selecting respondents are well-defined and justified.
- **Areas for Improvement:** The justification for focusing solely on urban Gen Z could be stronger. Discuss potential biases this might introduce and how they were mitigated.

** **


**Data Measurement:**

- **Strengths:** The use of established scales for measuring constructs is commendable. The questionnaire design and data collection process are well-explained.
- **Areas for Improvement:** Consider discussing potential biases in the questionnaire design. Were there any leading questions or ambiguities?

** **


**Sample Size:**

- **Strengths:** The sample size calculation is appropriate, and the use of a purposive sampling technique is justified.
- **Areas for Improvement:** The rationale for using a purposive sampling technique could be more critically discussed. Consider potential limitations of this approach.

**Statistical Methods:**

- **Strengths:** The use of SEM and other statistical techniques is appropriate for the analysis. The explanation of the methods is clear and detailed.
- **Areas for Improvement:** Some sections could benefit from more detail on why specific methods were chosen. Discuss any assumptions made and how they were addressed.

**4. Results**

**Reliability and Validity:**

- **Strengths:** The assessment of reliability and validity is thorough. The use of Cronbach's alpha, rhoa, and composite reliability is appropriate.
- **Areas for Improvement:** The discussion of reliability measures could be more critical. Were there any limitations in these measures?

**Structural Model:**

- **Strengths:** The evaluation of the structural model is well-explained. The use of bootstrapping and the reporting of path coefficients, R2, and Q2 values are commendable.
- **Areas for Improvement:** The presentation of results could be more concise. Some sections are overly detailed and could be streamlined.

**Mediation Analysis:**

- **Strengths:** The mediation analysis is well-conducted and clearly presented.
- **Areas for Improvement:** The interpretation of results could be more critical. Were there any limitations in the mediation analysis?

**Importance Performance Matrix Analysis (IPMA):**

- **Strengths:** The IPMA results are well-explained and provide valuable insights.
- **Areas for Improvement:** The discussion could be more critical. How do these results compare to existing literature?

**5. Discussion**

**Interpretation of Results:**

- **Strengths:** The discussion section effectively interprets the results in the context of existing literature. The findings are well-explained and supported by relevant studies.
- **Areas for Improvement:** The discussion could be more critical. Some interpretations feel overly positive without sufficient critical analysis.

**Theoretical and Managerial Implications:**

- **Strengths:** The implications are clearly stated and provide valuable insights for both theory and practice.
- **Areas for Improvement:** The discussion could be more critical. Are there any potential limitations or biases in these implications?

**Future Directions and Limitations**

**Future Research:**

- **Strengths:** The suggestions for future research are relevant and well-justified.
- **Areas for Improvement:** The suggestions could be more detailed. Consider discussing specific research questions or methodologies for future studies.

**Limitations:**

- **Strengths:** The limitations are clearly stated and appropriately acknowledged.
- **Areas for Improvement:** The discussion of limitations could be more critical. Consider discussing potential biases and how they might impact the results.

**Overall Comments**

- **Strengths:** The report is well-structured and comprehensive. It demonstrates a strong understanding of the topic and employs appropriate research methods. The writing is clear and concise, making the report easy to follow. The use of figures and tables enhances the presentation of the results.
- **Areas for Improvement:** The report could benefit from more critical analysis in several sections. Some areas are overly detailed and could be streamlined to enhance readability.
- Suggest relooking into each section above on the areas for improvement which will enhance better the report.

Is the work clearly and accurately presented and does it cite the current literature?

Yes

If applicable, is the statistical analysis and its interpretation appropriate?

Yes

Is the study design appropriate and is the work technically sound?

No

Are the conclusions drawn adequately supported by the results?

Yes

Reviewer Expertise:

Marketing, Digital Marketing , Consumer Behaviour, Entrepreneurship

I confirm that I have read this submission and believe that I have an appropriate level of expertise to confirm that it is of an acceptable scientific standard.

# Reviewer report: Reviewer response for version 1

**DOI:** 10.5256/f1000research.173012.r339089

**Published:** 2024-12-17

**Authors:** Meenakshi Sharma

**Affiliations:** 1 Birla Institute of Technology, Mesra, Ranchi, India

1. Detailed Explanation: The narrative style of the paper is scientific. The paper has a high degree of clarity and is focused. It contributes to the body of knowledge in the marketing domain, especially in the context of GenZ. It has been clearly articulated.
2. Figures and Tables: All figures and tables are in order and have an intext mentioned at appropriate places. Consistent with the paper's objective, the tables provide an overview of major findings.
3. Methodology: The methodology section is well drafted and captures the process appropriately. However, the authors are advised to explain the sample size estimation process in detail. This will strengthen the methodology section. There appears to be an error in the data measurement section. Relook at the “5-5 point likert scale”. In the pilot testing section “Similarity with the Influencer was below the threshold value of 0.7. In social science research, a construct with a Cronbach alpha above 0.6 in a pilot study could be retained as it is expected the increase with an increment in sample size. Hence the research team decided to retain the construct and explore it further during the final data analysis phase.” Authors are advised to include a  citation for this.
4. Data sharing: data is shared on Figshare and incorporated in the manuscript.
5. Discussion and Directions for future direction: The discussion section is well drafted and provides a comparison to previously published literature at appropriate places. A few grammatical errors are observed, Authors are advised to relook and incorporate corrections. Eg.. A sentence starts with “To address ……..”; Typo errors like 3.6….Cronbach alpha of all constructs of the study was above o.7 etc.

Authors are advised to take a relook at the paper and incorporate the suggestions presented above.

 The paper maybe accepted for indexing as it is written well and contributes to the body of knowledge of management science.

Is the work clearly and accurately presented and does it cite the current literature?

Yes

If applicable, is the statistical analysis and its interpretation appropriate?

Yes

Are all the source data underlying the results available to ensure full reproducibility?

Yes

Is the study design appropriate and is the work technically sound?

Yes

Are the conclusions drawn adequately supported by the results?

Yes

Are sufficient details of methods and analysis provided to allow replication by others?

Yes

Reviewer Expertise:

Marketing, Green banking, Sensory marketing

I confirm that I have read this submission and believe that I have an appropriate level of expertise to confirm that it is of an acceptable scientific standard.

# Response

**Published:** 2025-01-22

**Authors:** smitha nayak

**Affiliations:** Manipal University, India

Dear Reviewer, 

 Thank you for your observations and feedback on the manuscript. 

 I acknowledge the typo errors in the document and have included the corrections in the new version of the manuscript. 

 As far as the Cronbach Alpha goes, in social science research if the values are in the range of 0.5 to 0.7, it is still recommended to proceed with the scale as the assumption is that a bigger sample size will normalize this when the final data collection is undertaken. 

 I am uploading a new version of the manuscript after refining it with your suggestions. 

 Thank you again. 

# Reviewer report: Reviewer response for version 1

**DOI:** 10.5256/f1000research.173012.r339092

**Published:** 2024-11-27

**Authors:** Yoesoep Edhie Rachmad

**Affiliations:** 1 Education, United Nations Foundation (Ringgold ID: 50472), District of Columbia, Washington, USA; 2 Universal Institute of Professional Management, Shah Alam, Malaysia

Submission ID     : **2024_IJWOE-237645**

 Title                     :  Does Brand Attitude Complement Influencer Credibility in Shaping Purchase Intention of Indian GenZ Consumers?

 Authors               :  Pranav Vilas Chavare, Smitha Nayak, Ramona Birau, Varalakshmi Alapati.

 Journal                 : F1000Research 2024, 13:1343 Last updated: 13 NOV 2024

 Dear Authors,

  Thank you for your submission of the manuscript titled "Does Brand Attitude Complement Influencer Credibility in Shaping Purchase Intention of Indian GenZ Consumers?" This study offers a valuable and timely contribution to the field of influencer marketing, particularly in the context of Generation Z consumers in India. The research methodology, findings, and theoretical implications are well-articulated. However, I would like to suggest a few areas for improvement to further enhance the manuscript’s clarity, impact, and contribution.

**Feedback and Suggestions**

**Clarity in Presentation**

1. **Detailed Explanations:**

While the methodology section is robust, additional explanations regarding the choice of a five-point Likert scale and its appropriateness in this context would enhance understanding. Consider including a justification for its use over other scales.

1. **Figures and Tables:**

Some figures and tables, such as the structural model and hypothesis testing results, could benefit from clearer captions or legends. For example, explicitly defining significance levels (e.g., p values) directly in the table or figure notes would improve readability.

**Data Accessibility and Reproducibility**

1. **Data Sharing:**

It is commendable that the dataset is available on Figshare under a Creative Commons license. However, it may be beneficial to provide an appendix or a supplementary section with detailed instructions on how to access and use the dataset for replication studies.

**Discussion and Implications**

1. **Comparative Context:**

The discussion section could be expanded to include comparisons with similar studies in other regions or demographic groups. This would contextualize the findings and highlight their generalizability.

1. **Practical Applications:**

Including examples of how brands or marketers can apply these findings to develop effective influencer strategies would increase the manuscript's practical utility.

**Future Research Directions**

1. **Content and Product Categories:**

The study would benefit from a clearer acknowledgment of its limitations regarding generalizations across different content types or product categories. Suggestions for future research in these areas could provide valuable guidance for subsequent studies.

**Minor Corrections**

1. **Consistency in Terms:**

Ensure all technical terms are consistently defined throughout the manuscript. For instance, maintaining uniform terminology for constructs like “trust,” “expertise,” and “credibility” will aid comprehension.

1. **Grammar and Typographical Errors:**

A few minor typographical errors were noted. A careful review of the manuscript will help ensure overall accuracy and readability.

 Overall, this manuscript represents a significant contribution to the literature on influencer marketing and consumer behavior. The innovative approach and detailed analysis are commendable, and I am confident that with these refinements, the paper will achieve even greater impact.

 Thank you for the opportunity to review this work. I look forward to the revised version.

 Best regards,

**Prof. Dr. Yoesoep Edhie Rachmad**

 Reviewer

 Submission ID     : **2024_IJWOE-237645**

 Title                     :  Does Brand Attitude Complement Influencer Credibility in Shaping Purchase Intention of Indian GenZ Consumers?

 Authors               :  Pranav Vilas Chavare, Smitha Nayak, Ramona Birau, Varalakshmi Alapati.

 Journal                 : F1000Research 2024, 13:1343 Last updated: 13 NOV 2024

 Dear Editor,

  The manuscript titled "Does Brand Attitude Complement Influencer Credibility in Shaping Purchase Intention of Indian GenZ Consumers?" presents a compelling study that contributes significantly to the growing field of influencer marketing and its impact on consumer behavior, specifically within the context of Generation Z in India. While the research demonstrates robust methodology and meaningful findings, there are a few areas that I would recommend addressing to enhance the overall clarity and impact of the manuscript:

**Key Points for Consideration**

1. **Clarity and Accessibility in Methodology:**

The study employs PLS-SEM to test hypotheses effectively. However, for a broader audience, providing additional context or illustrative examples regarding the structural model and key metrics (e.g., significance thresholds) would increase clarity and accessibility.

  

1. **Data Availability and Reproducibility:**

While the authors commendably provide access to the dataset on Figshare, it may be beneficial to suggest including explicit guidance or links in an appendix to facilitate easier replication by future researchers.

  

1. **Discussion of Findings and Generalizability:**

The discussion section could be expanded to compare the findings with similar research across different demographics or industries. Highlighting potential applications of the study's insights in other regions or sectors would underscore its broader relevance.

  

1. **Practical Implications:**

The paper makes significant theoretical contributions. To balance this, elaborating on how brands can implement these findings to design effective influencer campaigns could enhance the manuscript’s practical utility.

  

1. **Future Research Directions:**

The limitations section briefly mentions opportunities for future work. However, more detailed suggestions, such as examining content types or distinguishing between macro- and micro-influencers, would provide valuable guidance for subsequent research in this field.

**Overall Recommendation**

 The manuscript offers a valuable addition to the literature on influencer marketing and consumer behavior. Addressing the points above through minor revisions would further strengthen its impact and readability.

 Thank you for the opportunity to review this manuscript. I look forward to seeing its refined version.

 Best regards,

**Prof. Dr. Yoesoep Edhie Rachmad**

 Reviewer

 Submission ID     : **2024_IJWOE-237645**

 Title                     :  Does Brand Attitude Complement Influencer Credibility in Shaping Purchase Intention of Indian GenZ Consumers?

 Authors               :  Pranav Vilas Chavare, Smitha Nayak, Ramona Birau, Varalakshmi Alapati.

 Journal                 : F1000Research 2024, 13:1343 Last updated: 13 NOV 2024

  I recommend **Minor Revisions** for the manuscript titled **"** **Does Brand Attitude Complement Influencer Credibility in Shaping Purchase Intention of Indian GenZ Consumers?".**

**Justification for Recommendation**:

  

1. **Innovative Approach:**

The manuscript provides an insightful examination of influencer credibility and its impact on purchase intention, particularly in the context of Generation Z in India. The integration of trust, expertise, and similarity in assessing influencer credibility adds a significant contribution to the literature on consumer behavior.

  

1. **Clarity and Detail:**

The study uses advanced methodologies such as PLS-SEM, which are well-documented. However, the manuscript would benefit from additional intuitive examples or explanations for readers less familiar with this approach. Adding more visuals or diagrams, particularly in the methodology section, could enhance clarity.

  

1. **Data Accessibility:**

While the dataset is made publicly available through Figshare, the authors should provide additional guidance or references for accessing and utilizing the data to support reproducibility.

  

1. **Discussion and Practical Implications:**

The discussion section is thorough but could be expanded with a stronger emphasis on practical applications. For instance, the authors could elaborate on how brands can leverage the findings to design effective influencer marketing campaigns.

  

1. **Minor Corrections:**

Some typographical errors and inconsistencies in the use of technical terms were noted. Ensuring uniformity in terminology and addressing these minor errors will improve the manuscript's readability.

**Conclusion:**

 This manuscript is a valuable contribution to the understanding of influencer marketing and consumer behavior. The proposed revisions are minor and intended to enhance the manuscript's clarity, practical relevance, and broader impact.

 I look forward to reviewing the revised version.

 Best regards,

**Prof. Dr. Yoesoep Edhie Rachmad**

 Reviewer

Is the work clearly and accurately presented and does it cite the current literature?

Yes

If applicable, is the statistical analysis and its interpretation appropriate?

Yes

Are all the source data underlying the results available to ensure full reproducibility?

Yes

Is the study design appropriate and is the work technically sound?

Yes

Are the conclusions drawn adequately supported by the results?

Yes

Are sufficient details of methods and analysis provided to allow replication by others?

Yes

Reviewer Expertise:

Yes, I consider aspects of this article to be of major significance.Justification:The study provides critical insights into the mechanisms by which influencer credibility and brand attitude shape purchase intentions, particularly among Gen Z consumers in a rapidly digitizing market like India. This demographic and geographical focus offers unique value in understanding emerging consumer behaviors.Why It Is Significant:Practical Implications:The findings can guide brands and marketers in designing more effective influencer strategies, especially in leveraging trust and expertise over attractiveness.Theoretical Contribution:By identifying the mediating role of brand attitude, the study advances existing frameworks like the Theory of Reasoned Action, adding depth to literature on influencer marketing and consumer behavior.Emerging Market Focus:The focus on Gen Z in India highlights evolving trends in an underrepresented region, providing valuable insights that could inform both academic and industry strategies globally.The work bridges theoretical and practical gaps, making it a substantial addition to the field of marketing research.

I confirm that I have read this submission and believe that I have an appropriate level of expertise to confirm that it is of an acceptable scientific standard.

# Response

**Published:** 2024-12-02

**Authors:** smitha nayak

**Affiliations:** Manipal University, India

1. While the methodology section is robust, additional explanations regarding the choice of a five point likert scale.

 Response : Changes have been incorporated. As these scales were originally developed and designed to be measured on 5 point likert scale, the same has been adopted in the manuscript.

 2. Data Sharing:

 It is commendable that the dataset is available on Figshare under a Creative Commons license. However, it may be beneficial to provide an appendix or a supplementary section with detailed instructions on how to access and use the dataset for replication studies.

 Response: This statement has been included in the “Underlying Data“ section of the. The underlying data of the manuscript can be accessed on Figshare by clicking on this link

 3. Including examples of how brands or marketers can apply these findings to develop effective influencer strategies would increase the manuscript's practical utility.

 Response: The same has already been addressed in the theoretical and managerial implication sections of the manuscript.

 4. Content and Product Categories:

 The study would benefit from a clearer acknowledgment of its limitations regarding generalizations across different content types or product categories. Suggestions for future research in these areas could provide valuable guidance for subsequent studies. 

 Response has been incorporated.

 5. Manuscript has been rechecked for Consistency, Typographical errors and Grammatical errors.
